# Effectiveness of the AZD1222 vaccine against COVID-19 hospitalization in Europe: final results from the COVIDRIVE test-negative case–control study

**DOI:** 10.1093/eurpub/ckae219

**Published:** 2025-01-21

**Authors:** Leonie de Munter, Wilhelmine Meeraus, Akshat Dwivedi, Marianna Mitratza, Chloé Wyndham-Thomas, Lucy Carty, Mario Ouwens, Wendy Hartig-Merkel, Laura Drikite, Griet Rebry, Irma Casas, Ainara Mira-Iglesias, Giancarlo Icardi, Susana Otero-Romero, Sebastian Baumgartner, Charlotte Martin, Xavier Holemans, Gerrit Luit ten Kate, Kaatje Bollaerts, Sylvia Taylor

**Affiliations:** P95 Epidemiology and Pharmacovigilance, Leuven, Belgium; Medical Evidence, Vaccines & Immune Therapies, BioPharmaceuticals Medical, AstraZeneca, Cambridge, United Kingdom; P95 Epidemiology and Pharmacovigilance, Leuven, Belgium; P95 Epidemiology and Pharmacovigilance, Leuven, Belgium; P95 Epidemiology and Pharmacovigilance, Leuven, Belgium; Medical and Payor Statistics, BioPharmaceuticals Medical, AstraZeneca, Cambridge, United Kingdom; Medical and Payor Statistics, BioPharmaceuticals Medical, AstraZeneca, Mölndal, Sweden; P95 Epidemiology and Pharmacovigilance, Leuven, Belgium; P95 Epidemiology and Pharmacovigilance, Leuven, Belgium; P95 Epidemiology and Pharmacovigilance, Leuven, Belgium; Preventive Medicine Department, Germans Trias i Pujol University Hospital, Badalona, Spain; Autonomous University of Barcelona, Bellaterra, Spain; Germans Trias i Pujol Research Institute (IGTP), Badalona, Spain; Vaccine Research Department, Fundación para el Fomento de la Investigación Sanitaria y Biomédica (FISABIO) de la Comunitat Valenciana, Salud Pública, Valencia, Spain; Biomedical Research Consortium of Epidemiology and Public Health (CIBER-ESP), Instituto de Salud Carlos III, Madrid, Spain; Interuniversity Research Centre on Influenza and Other Transmissible Infections (CIRI-IT), Genoa, Italy; Department of Health Sciences, University of Genoa, Genoa, Italy; Servicio de Medicina Preventiva y Epidemiología, Hospital Universitari Vall d’Hebron, Vall d’Hebron Barcelona Campus Hospitalari, Barcelona, Spain; Fourth Medical Department with Infectious Diseases and Tropical Medicine, Klinik Favoriten/Kaiser-Franz-Josef Hospital, Vienna, Austria; Infectious Diseases Department, Centre Hospitalier Universitaire Saint-Pierre, Brussels, Belgium; Infectious Diseases Department, Grand Hôpital de Charleroi, Charleroi, Belgium; General Internal Medicine, Infectious Diseases & Tropical Medicine, University Hospital Antwerp, Edegem, Belgium; P95 Epidemiology and Pharmacovigilance, Leuven, Belgium; Medical Evidence, Vaccines & Immune Therapies, BioPharmaceuticals Medical, AstraZeneca, Cambridge, United Kingdom

## Abstract

Marketing authorization holders of vaccines typically need to report brand-specific vaccine effectiveness (VE) to the regulatory authorities as part of their regulatory obligations. COVIDRIVE (now id. DRIVE) is a European public–private partnership for respiratory pathogen surveillance and studies of brand-specific VE with long-term follow-up. We report the final VE results from a two-dose primary series AZD1222 (ChAdOx1 nCoV-19) vaccine schedule in ≥18-year-old individuals not receiving boosters. Patients (*N* = 1,333) hospitalized with severe acute respiratory infection at 14 hospitals in Austria, Belgium, Italy, and Spain were included in the test-negative case–control study in 2021–2023. Absolute VE was calculated using generalized additive model (GAM), generalized estimating equation (GEE), and spline-based area under the curve (AUC, measuring VE up to 6 months after the last dose of AZD1222). Overall VE (against coronavirus disease 2019 [COVID-19] hospitalization) of an AZD1222 primary series was estimated as 65% using GEE (95% confidence interval [CI]: 52.9–74.5), and 69% using GAM (95% CI: 50.1–80.9) over the 22-month study period (comparator group: unvaccinated patients). The AUC of the spline-based VE estimate was 74.1% (95% CI: 60.0–88.3). VE against hospitalization in study participants who received their second AZD1222 dose 2 months or less before hospitalization was 86% using GEE (95% CI: 77.8–91.4), 93% using GAM (95% CI: 67.2–98.6). During this study period, where mainly the severe acute respiratory syndrome coronavirus 2 Omicron variant was circulating, a two-dose primary series AZD1222 vaccination conferred protection against COVID-19 hospitalization up to at least 6 months after the last dose.

## Introduction 

The European Medicines Agency (EMA) granted a conditional marketing authorization for the AstraZeneca coronavirus disease 2019 (COVID-19) vaccine (AZD1222, ChAdOx1 nCoV-19) on 29 January 2021. Full marketing authorization was granted on 31 October 2022. Although AZD1222 was underused relative to other COVID-19 vaccines in Europe, the marketing authorization holder (MAH) AstraZeneca committed to monitoring vaccine effectiveness (VE) in the real world.

This article presents the final study results of the AstraZeneca COVIDRIVE study. COVIDRIVE (now id. DRIVE) is a public–private partnership, established in November 2020, to address the joint need to monitor real-world COVID-19 VE against hospitalization in Europe. It enables MAHs to report brand-specific COVID-19 VE as part of their regulatory obligations. In August 2023, we published the interim results from the AstraZeneca COVIDRIVE study, which focused on a population of vaccinated, but unboosted individuals [1]. Here, we report the final results from the AstraZeneca COVIDRIVE study on the real-world VE and durability of a two-dose AZD1222 primary series against COVID-19 hospitalization in four European countries (Austria, Belgium, Italy, and Spain) in unboosted individuals. This study focuses on an understudied COVID-19 vaccine platform and is unique because of its long follow-up period in a broader European context. It is thus an important addition to other existing reports of brand-specific AZD1222 real-world VE, which were mostly conducted in single countries and with shorter periods of follow-up [2–9].

## Methods

Methods have been previously described [1]. In brief, this AstraZeneca COVIDRIVE study had a multi-country, multi-centre, hospital-based, test-negative case–control (TNCC) design. Study participants were required to be aged ≥18 years and hospitalized with severe acute respiratory infection (SARI). SARI was defined based on a suspicion of respiratory infection with onset within the last 14 days prior to hospital admission and with at least one of the following symptoms: cough, fever, shortness of breath, or sudden onset of anosmia, ageusia, or dysgeusia [10]. Study participants were admitted between 1 May 2021 and 1 March 2023 to 1 of 14 participating hospitals in Austria, Belgium, Italy, and Spain. Participants were required to be eligible for COVID-19 vaccination prior to hospitalization. Test-positive cases were defined as patients who tested positive in at least one reverse transcriptase polymerase chain reaction (RT-PCR) assay on a sample taken between 14 days prior to and up to 24 h after hospital admission with SARI. Test-negative controls were defined as patients who tested negative in all RT-PCR tests on samples taken between 14 days prior to and up to 24 h after hospital admission with SARI. The respiratory swab test results were available from hospital medical records.

The exposed group was defined as patients fully vaccinated with two doses of AZD1222 (as a primary series), which had been administered 4–12 weeks apart, with the last dose given at least 14 days before symptom onset. Patients who received their second vaccination later than 14 days before symptom onset were defined as only partially vaccinated with one dose of AZD1222 and were excluded from analyses. Likewise, we excluded patients who were fully vaccinated and had additionally received a booster dose prior to symptom onset. The unexposed group was defined as patients who had never been vaccinated with any vaccine brand against COVID-19 prior to symptom onset. Sources for exposure ascertainment included vaccination registries, medical records, and vaccination cards, depending on the country and hospital.

Absolute VE against COVID-19 hospitalization was estimated as VE = (1–OR) × 100%, where OR denotes the odds ratio calculated as the ratio of the odds of having been fully vaccinated with two doses of AZD1222 among SARS-CoV-2 test-positive cases to the odds of vaccination among SARS-CoV-2 test-negative controls. All VE estimates were adjusted for symptom onset date, age, sex, and number of chronic conditions and were calculated for the overall study period and by time since last dose of AZD1222 (1 month was considered equal to 4 weeks).

As in the previously published interim analysis [1], VE was calculated using an additive fixed-effects logistic regression model [generalized additive model (GAM)]. Restricted maximum likelihood estimation (REML) was used to estimate the coefficients of the logistic regression and to select an optimal smoothing parameter for the spline effects (i.e. age and symptom onset). In the final analysis, VE was also calculated using generalized estimating equation (GEE) and a spline-based area under the curve (AUC). While both GEE and GAM are one-stage pooling methods, they differ in interpretation: VE estimates from the GEE model are conditional on the covariates but averaged over the participating hospitals (population-averaged estimates), whereas estimates from the GAM model are conditional on the covariates as well as on the participating hospitals. To enhance model robustness for GEE, a penalized generalized estimating equations (PGEE) approach with a ridge penalty on the cubic regression B-spline coefficients was adopted [11, 12]. The optimal penalty parameter was determined via five-fold cross-validation [13]. The degrees of freedom for the spline terms were set to 15 for symptom onset date and 3 for age. The Liang and Zeger method for fitting logistic regression models was utilized, assuming an exchangeable correlation structure to address potential within-cluster homogeneity [14]. Robust standard errors were calculated using the sandwich estimator [15]. GEE was pre-specified as primary analysis. The spline-based AUC was used as a secondary alternative measure of the VE. The AUC was calculated over an interval representing the period of 2 weeks to 6 months since the last dose of AZD1222 and was rescaled by dividing by the length of this interval. The point estimate was obtained by using the trapezoidal rule with 1000 equidistant points, and a 95% confidence interval (CI) was obtained using the Delta method.

Multiple, pre-planned sensitivity analyses were conducted, including a leave-one-out procedure to understand the impact of potential outlying and influential study sites on the VE and a quantitative bias analysis using evidence-based estimates to estimate bias due to missing information on prior severe acute respiratory syndrome coronavirus 2 (SARS-CoV-2) infection.

## Results

**Figure 1.**
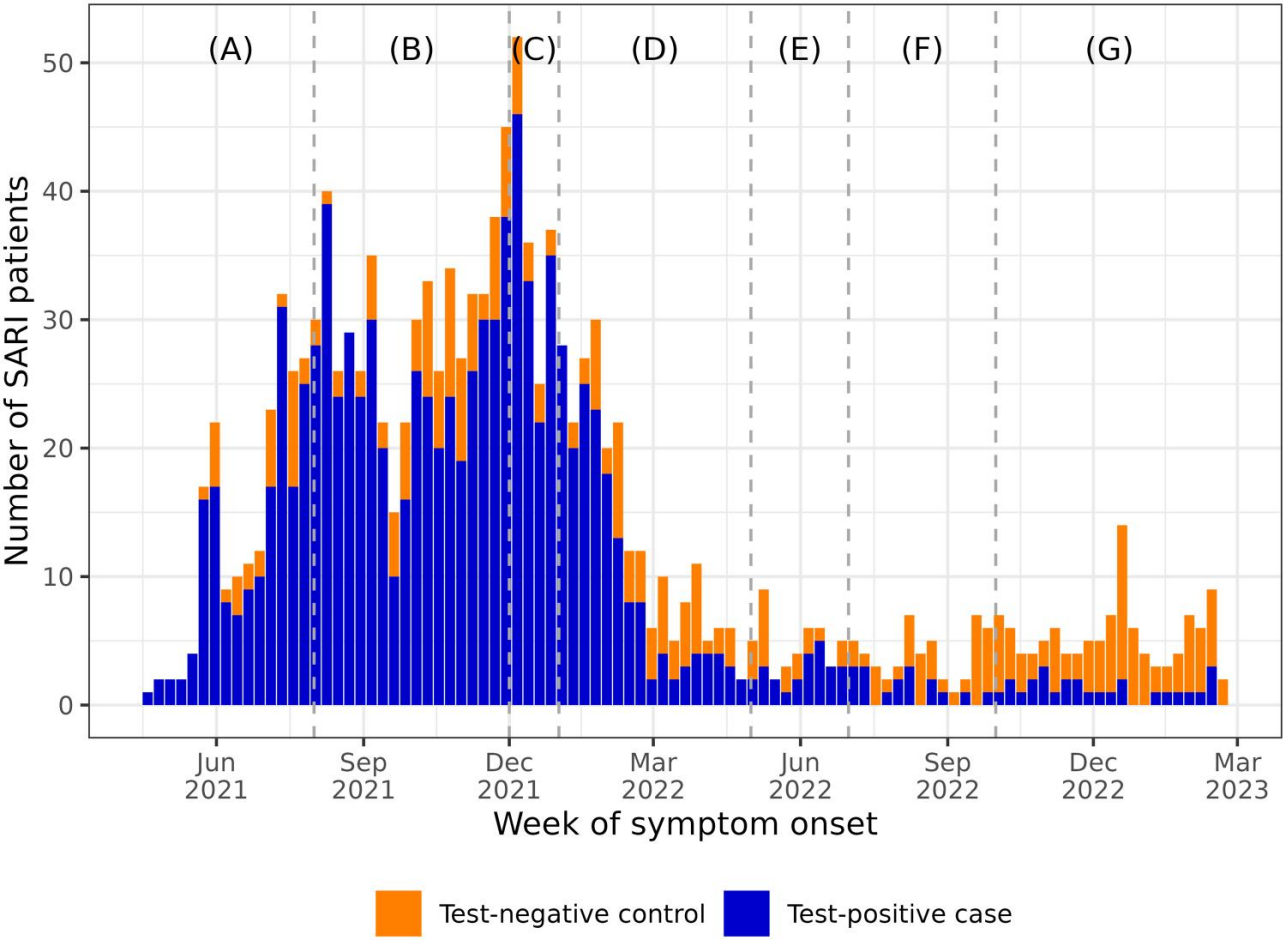
Timing of SARI symptom onset and SARS-CoV-2 variant predominance in the AstraZeneca COVIDRIVE study. Dashed lines indicate the boundaries between variant predominance periods: (A) 1 May 2021–31 July 2021 (Gamma, Alpha, Delta mixed); (B) 1 August 2021–30 November 2021 (Delta predominant); (C) 1 December 2021–31 December 2021 (Delta and Omicron BA.1 mixed); (D) 1 January 2022–30 April 2022 (Omicron BA.1/BA.2); (E) 1 May 2022–30 June 2022 (Mixed Omicron BA.2, BA.4, BA.5); (F) 1 July 2022–30 September 2022 (Omicron BA.5 predominant); (G) 1 October 2022–1 March 2023 (Mixed Omicron BA.5, BQ.1, BA.2.75, XBB).

Recruitment of study participants was completed on 1 March 2023 (Fig. 1). In total, 1,333 SARI patients (1,001 cases, 332 controls), recruited between 1 May 2021 and 1 March 2023, were included in the final analysis. Compared to the interim analysis, which included 761 study participants recruited over a 15-month period between 1 June 2021 and 5 September 2022 [1], the final analysis included more study participants, six additional months of follow-up, one additional study site, and retrospective data from three study sites. During the study period, the SARS-CoV-2 Delta variant was predominant for the first 7 months, with Omicron subvariants predominant for the remaining 15 months (Fig. 1).

Overall, study participant characteristics in the final analysis were comparable to those in the interim analysis. The mean age among test-negative controls and test-positive cases was 60.8 years [standard deviation (SD): 17.9 years] and 56.5 years (SD: 17.6 years), respectively. In total, 37% (*n* = 496) of participants were ≥65 years old, 44% (*n* = 585) were females, and 61% (*n* = 810) had at least one reported chronic condition, including 6% (*n* = 83) with immunodeficiency. SARI symptom onset occurred mostly between July 2021 and December 2021 (60%, *n* = 800) (Table 1). Of the 1,333 SARI patients, 148 patients were fully vaccinated with two doses of AZD1222 (and no boosters), and 1,185 were unvaccinated. In total, 89% (*n* = 132) of the fully vaccinated patients received their last dose of AZD1222 in June and July 2021 (Table 1). Fully vaccinated patients were older (median age of 64 [interquartile range (IQR): 61, 68] and 57 [IQR: 43, 71], respectively) and reported higher numbers of comorbidities (at least three chronic conditions reported in 31% and 18%, respectively) compared to unvaccinated patients.

**Table 1.** Characteristics of test-negative controls and test-positive cases hospitalized with SARI and included in the final analysis of the AstraZeneca COVIDRIVE study

	Total, *N* = 1,333	Test-negative controls, *n* = 332	Test-positive cases, *n* = 1,001
**Country, *n* (%)**			
Austria	378	4 (1%)	374 (37%)
Belgium	254	40 (12%)	214 (21%)
Italy	155	52 (16%)	103 (10%)
Spain	546	236 (71%)	310 (31%)
**Sex, *n* (%)**			
Female	585	133 (40%)	452 (45%)
Male	748	199 (60%)	549 (55%)
**Age**			
Years, mean (SD)	57.6 (17.8)	60.8 (17.9)	56.5 (17.6)
Years, median (lower, upper quartile)	59 (44, 70)	63 (49, 72)	57 (43, 69)
18–49 y, *n* (%)	439	84 (25%)	355 (35%)
50–64 y, *n* (%)	398	90 (27%)	308 (31%)
≥65 y, *n* (%)	496	158 (48%)	338 (34%)
**Number of chronic conditions, *n* (%)**			
0	523	86 (26%)	437 (44%)
≥1	810	246 (74%)	564 (56%)
**Month and year of SARI symptom onset, *n* (%)**			
April–June 2021	84	12 (4%)	72 (7%)
July–September 2021	348	42 (13%)	306 (31%)
October–December 2021	452	77 (23%)	375 (37%)
January–March 2022	215	53 (16%)	162 (16%)
April–June 2022	65	26 (8%)	39 (4%)
July–September 2022	53	33 (10%)	20 (2%)
October–December 2022	76	57 (17%)	19 (2%)
January–March 2023	40	32 (10%)	8 (1%)
**SARI level of severity, *n* (%)**			
Hospital admission without ICU admission and without in-hospital death	1,008 (75.6%)	304 (91.6%)	704 (70.3%)
ICU admission without in-hospital death	192 (14.4%)	14 (4.2%)	178 (17.8%)
In-hospital death	133 (10.0%)	14 (4.2%)	119 (11.9%)
**Length of SARI stay in hospital**			
Days, mean (SD)	12.1 (19.8)	7.7 (7.5)	13.6 (22.2)
Days, median (lower, upper quartile)	8.0 (4.0, 13.0)	6.0 (3.0, 9.0)	8.0 (5.0, 15.0)
≤3 days, *n* (%)	272 (21.8%)	98 (31.9%)	174 (18.6%)
4–7 days, *n* (%)	303 (24.3%)	88 (28.7%)	215 (22.9%)
≥8 days, *n* (%)	670 (53.8%)	121 (39.4%)	549 (58.5%)
**Prior SARS-CoV-2 infection, *n* (%)**			
No known/reported prior infection	970	173 (52%)	797 (80%)
Yes—clinically diagnosed	3	1 (0%)	2 (0%)
Yes—laboratory-confirmed	30	20 (6%)	10 (1%)
Missing	330	138 (42%)	192 (19%)
**AZD1222 vaccine exposure**			
Unvaccinated, *n* (%)	1185	262 (79%)	923 (92%)
Fully vaccinated with two doses, *n* (%)	148	70 (21%)	78 (8%)
Days since last dose, range	14–628	21–570	14–628
Days since last dose, median (lower, upper quartile)	142 (102, 166)	122 (82, 183)	152 (121, 164)

ICU, intensive care unit; *n*/*N*, number; SARS-CoV-2, severe acute respiratory syndrome coronavirus 2; y, years.

Confounder-adjusted VE against COVID-19 hospitalization (measured over the 22-month study period) was 69.2% (95% CI: 50.1–80.9) using GAM and 65.4% (95% CI: 52.9–74.5) using GEE (Table 2). As the study period increased from 15 months for the interim analysis to 22 months for the final analysis, so did the maximum participant follow-up time after the last dose (from 439 [1] to 628 days [Table 1]). The AUC of the spline-based VE estimate (measuring effectiveness for up to 6 months after the last dose of AZD1222) was calculated as 74.1% (95% CI: 60.0–88.3). Among study participants who received their second AZD1122 dose ≤2 months prior to hospitalization, VE was 93.2% (95% CI: 67.2–98.6) using GAM and 86.2% (95% CI: 77.8–91.4) using GEE (Table 2). VE estimates decreased with increasing time since last dose of AZD1222, though CIs were wide, in particular when exceeding 6 months after the last dose (Table 2). These final results are in line with the previously published interim results [1]. Although VE heterogeneity was observed at study site level, the overall VE point estimates from the leave-one-out procedure differed by no more than 1.6%. Even though information on prior SARS-CoV-2 infection was missing for many study participants, results from the quantitative bias analysis did not differ substantially from the main VE results (bias-corrected VE: 68%) [16].

**Table 2.** Effectiveness of a two-dose primary series of AZD1222, by time since last dose and overall, estimated using GEE and GAM methods in the AstraZeneca COVIDRIVE study

		Test-negative controls	Test-positive cases	Time since last dose in days	VE % (95% CI)^a^	
Time since last dose	Exposure					
		*N* (%)	*N* (%)	Median (lower, upper quartile)	GEE	GAM
≤2 months	Vaccinated	8 (62%)	5 (38%)	42 (39, 48)	86.2%	93.2%
	Unvaccinated	262 (22%)	923 (78%)		(77.8; 91.4)	(67.2; 98.6)
>2 and ≤4 months	Vaccinated	23 (66%)	12 (34%)	93 (78, 103)	78.4%	82.7%
	Unvaccinated	262 (22%)	923 (78%)		(57.0; 89.2)	(55.2; 93.3)
>4 and ≤6 months	Vaccinated	20 (30%)	46 (70%)	146 (132, 159)	54.1%	57.1%
	Unvaccinated	262 (22%)	923 (78%)		(−1.9; 79.3)	(10.1; 79.6)
>6 and ≤8 months	Vaccinated	4 (27%)	11 (73%)	189 (174, 204)	36.2%	35.0%
	Unvaccinated	262 (22%)	923 (78%)		(−138.6; 82.9)	(−138.3; 82.3)
>8 and ≤10 months	Vaccinated	3 (50%)	3 (50%)	260 (257, 264)	21.2%	28.8%
	Unvaccinated	262 (22%)	923 (78%)		(−987.9; 94.3)	(−301.8; 87.4)
>10 and ≤12 months	Vaccinated	4 (100%)	0 (0%)	307 (298, 323)	NE^b^	NE^b^
	Unvaccinated	262 (22%)	923 (78%)			
>12 months	Vaccinated	8 (89%)	1 (11%)	485 (447, 549)	45.6%	36.5%
	Unvaccinated	262 (22%)	923 (78%)		(−268.6; 92.0)	(−476.6; 93.0)
All	Vaccinated	70 (47%)	78 (53%)	142 (102, 166)	65.3%	69.2%
	Unvaccinated	262 (22%)	923 (78%)		(52.9; 74.5)	(50.1; 80.9)

CI, confidence interval; GAM, generalized additive model; GEE, generalized estimating equation; VE, vaccine effectiveness.

a Adjusted for onset date, number of chronic conditions, sex, and age.

b Non-estimable VE due to zero breakthrough cases, with all test-positive cases occurring in unvaccinated individuals.

## Discussion

The final results from the AstraZeneca COVIDRIVE study showed that a two-dose primary series of AZD1222 maintained protection against COVID-19 hospitalization for up to at least 6 months. Compared with other European studies, VE estimates in this study were similar [9, 17], while in some cases slightly lower [18, 19]. However, direct comparisons to other studies are difficult due to differences in country setting, study period, study population, outcome definitions, and testing methods. In addition, differences in country-specific recommendations for COVID-19 vaccination, SARS-CoV-2 testing policies, infection rates, and characteristics of the population choosing to be vaccinated (or not) may have influenced the overall VE results. Estimates of COVID-19 VE may also vary between and within studies due to changes in circulating SARS-CoV-2 variants and due to increasing time since the last vaccine dose. The fact that the vast majority of study participants in this AstraZeneca COVIDRIVE study received their last dose of AZD1222 in June and July 2021 may explain the decreasing trend in VE over time that was observed in the final analysis. For example, this could indicate waning immunity or potentially reduced protection against Omicron compared to earlier predominant variants. Delta was the predominant variant during the first months of the study, while Omicron BA.1 emerged in December 2021 and rapidly became the predominant variant across the different participating countries for 4 months, followed by Omicron BA.2 and Omicron BA.5. In total, Omicron subvariants were predominant for 15 of the 22 months of the study period. Nonetheless, the prolonged protective effect of a two-dose primary series of the AZD1222 vaccine across SARS-CoV-2 genetic variants and time periods can be attributed to the polyfunctionality of the T-cell immune response, which induces a broad spectrum of memory function [20]. Polyfunctional CD4 T cells have been found to be well-maintained 6 months after the second vaccination [21], while the vast majority of CD4 and CD8 epitopes are conserved across multiple variants [22].

A key strength of this study was the use of the COVIDRIVE (now id. DRIVE) platform, which has shown to reliably deliver high-quality epidemiological studies through a sustainable European research network. As of June 2024, id. DRIVE has enrolled >15 000 SARI patients [23]. The platform enables the shared collection of real-world, regional-level data, which are of high value to public health for monitoring VE and to industry for post-authorization regulatory commitments. In addition, the TNCC design is an efficient study design frequently used for the evaluation of VE [24]. Compared to a classical case–control study, the TNCC design reduces potential selection biases (e.g. due to healthcare-seeking behaviour) and is suited to study rare outcomes. COVIDRIVE collected detailed information from medical records, patients, and healthcare providers. Furthermore, the study included hospitals from several European countries and thus has greater generalizability than a single-country study. However, the results should be interpreted in light of the study limitations. First, the number of SARI subjects eligible for the final analysis was limited, as most vaccinated adults in Europe received booster doses during the booster vaccine campaigns (using predominantly mRNA-based vaccines), thus making them ineligible for our analysis, which focused on unboosted adults. The study sample size was therefore limited, resulting in insufficient numbers to conduct stratified analyses (e.g. per period of predominant variant). Second, missing information on prior SARS-CoV-2 infection may be expected to cause unmeasured confounding, leading to reduced VE estimates. However, a sensitivity analysis demonstrated that the magnitude of potential bias was minimal, likely due to low levels of prior SARS-CoV-2 infection prior to primary series vaccination (90% of individuals received their second dose in June and July 2021). The high rates of missing information on prior COVID-19 diagnosis (and SARS-CoV-2 infection) prevented further investigation of unmeasured confounding and of the impact of depletion of susceptibles (i.e. when the unvaccinated population has a higher level of natural immunity due to prior SARS-CoV-2 infection) on our estimates of VE. Third, varying site start dates for enrolment and the differing country-specific recommendations for COVID-19 vaccination resulted in inter-site heterogeneity. However, VE estimates from sensitivity analyses excluding subjects from a specific hospital or hospital network did not differ substantially, indicating robustness of the overall VE estimates. Fourth, although all patients met the SARI case definition, and all were tested for SARS-CoV-2, it remains a small possibility that incidental COVID-19 cases (individuals who are hospitalized for reasons other than COVID-19) were included, potentially leading to an underestimation of VE [8].

The complex interplay of the time-related factors (e.g. SARS-CoV-2 epidemic waves, genetic SARS-CoV-2 variants, levels of natural immunity in the population, and the implementation of COVID-19 vaccination programmes) makes it challenging to disentangle waning vaccine immunity from potentially different VE against specific genetic variants. Nevertheless, we conclude that a two-dose primary series of AZD1222 conferred protection against COVID-19 hospitalization up to at least 6 months after the last dose. These overall estimates cover the entire study period of 22 months, and therefore different SARS-CoV-2 incidence rates and circulating variants.

## Data Availability

De-identified data that underlie the results reported in the text and tables may be obtained in accordance with the COVIDRIVE (now id. DRIVE) data access policy. Key points This real-world European study evaluated the long-term effectiveness of the AZD1222 (ChAdOx1 nCoV-19) vaccine against COVID-19 hospitalization during periods of Delta and Omicron variant predominance. A two-dose primary series of AZD1222 vaccine showed effectiveness of 65%–69% (with median time since last dose of 142 days and estimated using different methods). For those who received their second dose of AZD1222 two months or less prior to hospitalization, effectiveness was 86%–93% (depending on method for estimating VE) in preventing hospital admissions for SARI due to SARS-CoV-2. Despite possible lower effectiveness against Omicron, AZD1222 protected against hospitalization for up to at least 6 months post-second dose.
